# Exploring Two Honey Bee Traits for Improving Resistance Against *Varroa destructor*: Development and Genetic Evaluation

**DOI:** 10.3390/insects12030216

**Published:** 2021-03-03

**Authors:** Matthieu Guichard, Benoît Droz, Evert W. Brascamp, Adrien von Virag, Markus Neuditschko, Benjamin Dainat

**Affiliations:** 1 Agroscope, Swiss Bee Research Centre, Schwarzenburgstrasse 161, 3003 Bern, Switzerland; benoit.droz@agroscope.admin.ch (B.D.); adrien.vonvirag@agroscope.admin.ch (A.v.V.); markus.neuditschko@agroscope.admin.ch (M.N.); benjamin.dainat@agroscope.admin.ch (B.D.); 2 Wageningen University & Research Animal Breeding and Genomics, P.O. Box 338, 6700 AH Wageningen, The Netherlands; pim.brascamp@wur.nl

**Keywords:** *Apis mellifera*, *Recapping*, *Solidness*, *Varroa destructor*

## Abstract

**Simple Summary:**

Selection of honey bees requires traits which can be easily measured in the field by beekeepers. This is also the case for traits linked to honey bee resistance against the parasitic mite *Varroa destructor*. We therefore describe two new trait evaluation protocols, *‘Recapping’* and *‘Solidness’*, conceived to enable an easy evaluation of two putative colony resistance traits, recapping (i.e., opening and re-sealing) and solidness (i.e., amount of capped brood in a defined area) of worker brood, respectively. The hypothesis of this study is that higher levels of *‘Recapping’* and *‘Solidness’* could provide resistance to *V. destructor*. Repeatability and heritability of the two traits, as well as their phenotypic correlations with other colony traits were calculated, in order to investigate their potential for resistance selection. Both traits showed low repeatability between different measurements within each year. *‘Recapping’* had a low heritability and a negative correlation to hygienic behavior evaluated by the pin-test method. The heritability of *‘Solidness’* was moderate. The two traits did not show an association with *V. destructor* infestation levels. Further research is needed to confirm these results, as only a small number of colonies could be evaluated.

**Abstract:**

For the development of novel selection traits in honey bees, applicability under field conditions is crucial. We thus evaluated two novel traits intended to provide resistance against the ectoparasitic mite *Varroa destructor* and to allow for their straightforward implementation in honey bee selection. These traits are new field estimates of already-described colony traits: brood recapping rate (‘*Recapping*’) and solidness (‘*Solidness*’). ‘*Recapping’* refers to a specific worker characteristic wherein they reseal a capped and partly opened cell containing a pupa, whilst *‘Solidness’* assesses the percentage of capped brood in a predefined area. According to the literature and beekeepers’ experiences, a higher recapping rate and higher solidness could be related to resistance to *V. destructor*. During a four-year field trial in Switzerland, the two resistance traits were assessed in a total of 121 colonies of *Apis mellifera mellifera*. We estimated the repeatability and the heritability of the two traits and determined their phenotypic correlations with commonly applied selection traits, including other putative resistance traits. Both traits showed low repeatability between different measurements within each year. ‘*Recapping*’ had a low heritability (h^2^ = 0.04 to 0.05, depending on the selected model) and a negative phenotypic correlation to non-removal of pin-killed brood (r = −0.23). The heritability of ‘*Solidness*’ was moderate (h^2^ = 0.24 to 0.25) and did not significantly correlate with resistance traits. The two traits did not show an association with *V. destructor* infestation levels. Further research is needed to confirm the results, as only a small number of colonies was evaluated.

## 1. Introduction

*Varroa destructor* is still the main pest threatening *Apis mellifera* in many countries [1,2]. Currently implemented solutions, such as chemical treatments, are known to lack sustainability [3,4,5,6,7], leading to an urgent need to improve current strategies. The selection of resistant honey bees, which limit the reproduction or survival of *V. destructor* within the colony, is a strategy to decrease infestation levels and ultimately improve colony survival [8,9].

To select for resistance, chosen traits have to be closely and stably linked to resistance, they must be heritable, and for practical application, they should be easily assessable to beekeepers under field conditions in order to facilitate broad-scale selection success. For instance, hygienic behaviour towards dead brood is assessed with a pin test, which is an affordable test routinely performed by many European beekeepers. However, the correlation of the results of this test with *V. destructor* infestation levels is still under discussion [9,10,11]. On the other hand, most currently applied resistance traits are both time consuming and costly to assess [8]: for instance, the evaluation of suppressed mite reproduction requires the dissection of several hundred brood cells under a stereomicroscope [12], which can only be processed by a limited number of highly qualified beekeepers.

To support the selection against *V. destructor*, we derived two novel traits from existing phenotypes, namely recapping (*‘Recapping’*) and solidness (*‘Solidness’*). Recapping refers to a specific worker characteristic wherein a capped and partly opened cell containing a pupa is re-sealed. This mechanism has been suggested to disturb mite reproduction [13] and in some cases to lead to mites leaving the disturbed brood cell [14]. A recapping test was developed because the natural survival of multiple resistant honey bee populations is associated with a high recapping rate [13,15], suggesting its role in promoting colony resistance. However, the current protocols for evaluating recapping rates are very time consuming, as this process includes the opening of hundreds of individual cells [13,16]. To increase the applicability of this trait, we developed a simplified protocol (‘*Recapping*’) which allows for a cost- and time-effective evaluation of this trait. Brood solidness assesses the percentage of capped brood in a predefined area. Beekeepers in Europe often state that a solid brood pattern might be associated with a healthy brood and a low infestation rate, whereas others tend to attribute a lower solidness to a high level of Varroa Sensitive Hygiene in the colony. To the best of our knowledge no results have been published on the association between solidness and colony resistance. Hence, we evaluated solidness to verify beekeeper’s assumptions. To date, brood solidness has been assessed independently from resistance to *V. destructor* [17]; this protocol was therefore adapted as further described below (‘*Solidness’*) to better fit with the requirements of our study.

Over a time period of four years (2017–2020), the two resistance traits were evaluated in a total of 121 colonies of *A. m. mellifera* to calculate heritability estimates. Simultaneously, the repeatability between different measurements was calculated for each year. Further, we investigated the association between the currently applied traits and the two novel selection traits. For this, we calculated phenotypic correlations between all traits. Routinely evaluated traits not related to *V. destructor* were also included in the calculation of phenotypic correlations, in order to evaluate the relevance of the two new traits with regard to the current selection process of the beekeepers.

## 2. Materials and Methods

### 2.1. Colonies, General Management and Performance Testing Protocol

In the summer of 2016, an experimental apiary with *A. m. mellifera* colonies was established in Canton Bern, Switzerland. The queens heading these colonies were reared by the association mellifera.ch in the same year and were clipped after introduction. In 2018, the experimental apiary was re-located to Canton Fribourg, Switzerland, and in 2020, a second apiary was established in Canton Bern. All colonies were hosted in 12-frame Dadant-Blatt hives, with undrawn frames and supers added according to colony development. Swarming control was performed twice per week as long as swarming cells were found in the colonies. Colonies were re-queened in autumn, with daughter queens produced in summer. These queens were reared from tested colonies and, each year, all mated at official mating stations with *A. m. mellifera* drones reared by the association mellifera.ch. In 2017 and 2019, drones descended from one single paternal origin, i.e., queens of the drone-producing colonies were sisters, whereas in 2018 a pool of drone producing colonies of two different origins were used for mating.

From 2017 to 2020, a total of 121 colonies completed a performance testing protocol between spring (time of willow blooming) and summer (last honey harvest). The following numbers indicate the successful evaluated colonies per year: 6 in 2017, 29 in 2018, 39 in 2019, and 47 in 2020. The minimum, median and maximum number of tested daughters/dam queen were 2, 9, and 13, respectively. The two novel traits (described in detail below), as well as commonly applied selection traits routinely measured by beekeepers [18] were evaluated in this study; the evaluation protocols of all investigated traits, as well as a summary of observations, are provided in Table 1. This table also contains the names of each trait (in italics). Swarming queens were caught and later reintegrated into their original colonies. For those colonies, it was not possible to record *V. destructor* infestation and colony size in summer due to the disturbance caused by swarming. Before the start of the study and after each year, *V. destructor* infestation levels were standardized to a negligible rate (<50 mites) by applying acaricides to the colonies in summer and autumn (formic acid or oxalic acid after brood interruption) and later during winter (oxalic acid).

**Figure 1:**
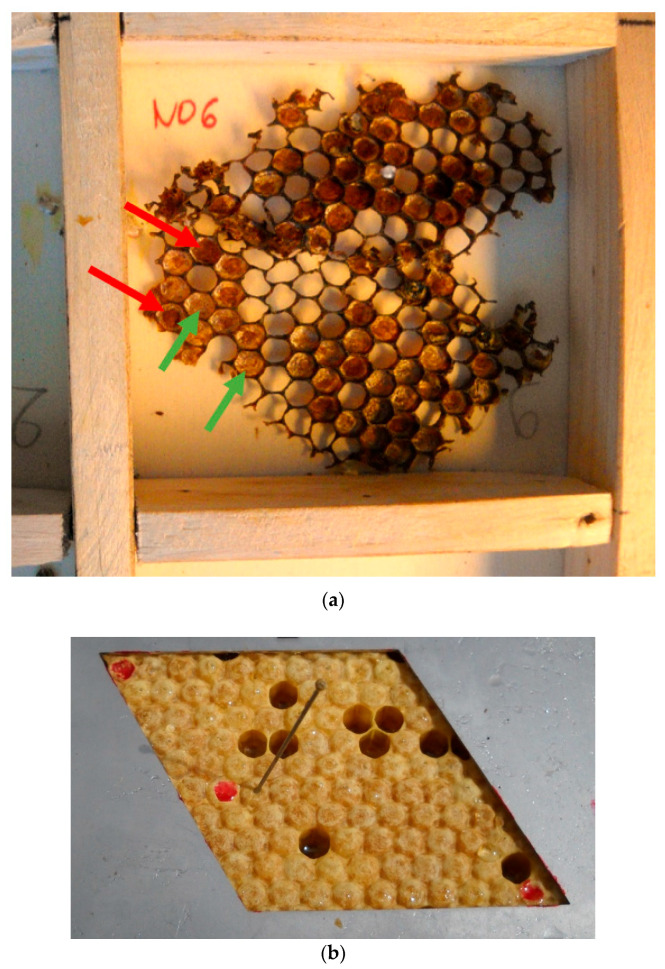
Illustration of ‘*Recapping*’ and ‘*Solidness*’ evaluation protocols. (**a**) Illustration of the evaluation procedure of the ‘*Recapping*’ trait. Cell cap samples are taken from a single colony and stored in their transportation box to be taken from the field to the evaluation desk. Some recapped cells are already clearly visible (e.g., those marked with red arrows) whereas others show an intact silk cocoon (e.g., those marked with green arrows). The sample is later taken out and examined under a stereomicroscope (×4) (**b**) Illustration of the evaluation procedure of the ‘*Solidness*’ trait. A pin-test was performed using an entomological pin. The upper red dot indicates the initial (non-pinned) pupa, then 50 pupae are pinned and the following (non-pinned) pupa gets another red dot (in the middle). Lowest red dot is used to place the template back to the test area when recording pin-test result. Between the upper two marks (delimitating 50 pin-killed pupae), 57 cells had to be checked to find 50 cells to pin (7 cells were empty). Here, observation value for ‘*Solidness*’ is 57.

For ‘*Recapping*’ (Figure 1a), about 100 worker cell caps were cut from a frame with a serrated knife. The sample was taken from a brood area next to emerging workers to target old brood (pupal stages). Indeed, in too recently capped brood (pre-pupal stages), the larvae have not spun their cocoon or workers may not have had time to inspect the cells. Therefore, the corresponding caps are not suitable for assessment. Furthermore, cells with emerging bees, which started opening the cell caps, were not included in the analysis.

The sampled cell caps were transported to the laboratory, where the total number of caps and the number of caps with missing silk (recapped) were counted under a stereomicroscope (x4).

‘*Solidness*’ (Figure 1b) was simultaneously evaluated with the pin test, which is performed on 50 capped cells (see Table 1). The total number of cells between the first and the last pinned pupae, including empty cells, was counted. The evaluated brood area was chosen according to the age of the brood for the pin test: it consisted of pink-eyed pupae, 6 days post-capping [19]. The proposed protocol for ‘*Solidness*’ was derived from the method described by [17] to enable trait recording along with the pin test, the latter currently being evaluated by Swiss beekeepers.

‘*Recapping*’, ‘*Solidness*’ and other selection traits (see Table 1) were recorded once every three weeks. However, the timespan between two consecutive measurements varied between 18 (measurement i = Friday and measurement i + 1 = Monday) and 25 (measurement i = Monday and measurement i + 1 = Friday) days, depending on weather conditions (e.g., colonies were not opened at rain or low temperatures to avoid colony losses). *‘Recapping’* was not evaluated in 2017.

### 2.2. Repeatability, Heritability, and Phenotypic Correlation Estimates

The repeatability of ‘*Recapping*’ and ‘*Solidness*’ was assessed by calculating Pearson’s correlations pairwise between the different measurements performed in 2018, 2019, and 2020. Too few colonies were measured for ‘*Solidness*’ in 2017 to calculate repeatability values for that year. It was not possible to calculate repeatability across years, as date and time intervals were not identical. In 2020, colonies were kept in two apiaries; hence, the location effect was included for this year.

Heritability estimate factors were calculated for all traits based on a Best Linear Unbiased Prediction (BLUP) model [24,25], which has also been applied in a recent study including *A. m. mellifera* colonies [18]. Briefly, for each colony, an input file was prepared that contained identification codes for the queen heading the colony, the mother of this queen, and the mother of the queens heading the drone-producing colonies used for the mating of the queen heading the tested colony. In addition, a performance file containing records for the different traits was prepared. Both files were generated in R [26]. Variance components associated with worker and queen effects were separately estimated with two linear models in ASReml software version 4.1.2132 (www.vsni.co.uk, accessed on 3 March 2021), which took into account the year/apiary combination (5 cases) as a fixed effect, as all colonies were located in a given apiary, equally managed and evaluated the same day. A joint estimation of worker and queen effects did not generate results. This was because the restricted size of the preliminary dataset did not enable convergence of the restricted maximum likelihood algorithm. Therefore, the variance for worker effect in the worker model to some extent includes effects of the queen as the pedigrees for workers and queen are partly the same, as is the residual variance. This in reverse is the case for the queen model. To facilitate interpretation of the results, observation data were not transformed prior to the analysis. Pairwise phenotypic correlations were calculated between all traits, as defined in Table 1, after correction of the observations for fixed effects from the model on worker effect. Correlation after correction for the queen effect showed the same results. Therefore, these results are not presented. The standard errors (SEs) associated with the correlations were calculated as follows:

$$\mathrm{SE}\;=\;\frac{1-r^{2}}{\sqrt{N-2}},$$

with r being Pearson’s correlation coefficient between both traits and *N* − 2 being the number of degrees of freedom associated with N colonies having observations for both traits (as recently used by [27]). The significance of Pearson’s correlation coefficients was tested using the cor.test function in R [26], with a confidence interval of 0.95.

## 3. Results

The repeatability for ‘*Recapping*’ and ‘*Solidness*’ is presented in Table 2. In general, both traits had pairwise correlations between repetitions below 0.30 (40 out of 59 correlations). The highest correlations were found recorded in 2020 for ‘*Solidness*’, with values up to 0.80, whilst measurements on the diagonal were not different from those off the diagonal.

The heritability estimates and phenotypic correlation results of all traits are summarised in Table 3. Due to either the small dataset or unidentified specificities of data, the Restricted Maximum Likelihood (REML) algorithm did not converge for two traits, ‘*Gentleness*’ and ‘*Colbroodgrowth*’. Therefore, these two traits were removed from the downstream analyses. The other traits had estimated heritabilities ranging from 0.01 to 0.72, many of them being theoretically compatible with selection. For ‘*Recapping*’, heritabilities (±SE) were estimated at 0.05 (0.24) and 0.04 (0.26) using models for queen and worker effects, respectively, whilst the same models estimated the heritability for ‘*Solidness’* at 0.25 (0.29) and 0.24 (0.26), respectively. The overall highest heritabilities were obtained for the traits ‘*Calmness*’, ’*Varroasummer*’, ‘*Hygfull*’ and ‘*Colbeessummer*’. Heritability estimates under the two different models (worker and queen) were generally comparable.

Some of the correlations between phenotypes corrected for fixed effects showed moderate-to-high values (up to 0.65). For ‘*Recapping*’, a negative correlation (r = −0.23) was found with ‘*Hygfull*’, whilst ‘*Solidness*’ was negatively correlated with the quantity of brood produced in spring as well as with the adult worker populations in both spring and summer (−0.24 to −0.28). Otherwise, positive correlations were identified between honey production and colony size traits, between *V. destructor* infestation rates and colony size and between ‘*Hygfull*’ and ‘*Colbeessummer*’. A positive correlation was found between the number of untouched pinned cells (‘*Hygfull*’) and *V. destructor* infestation in summer (‘*Varroasummer*’) (0.32 (0.10)). A negative correlation was symmetrically obtained between the rate of cleared cells (‘*Hygempty*’) and ‘*Varroasummer*’ (−0.26 (0.11)).

## 4. Discussion

In this study, we evaluated two novel traits, ‘*Recapping*’ and ‘*Solidness*’, in an experimental *A. m. mellifera* population. Beekeepers can easily measure both traits as part of routine colony testing with minimal additional costs.

The repeatability of the two traits was relatively low in 2018–2020, and some consecutive measurements were even negatively correlated. This result reveals that it was not possible to obtain standardised measurements, due to high observed variations of the traits across the season. In 2020, the repeatability of ‘*Solidness*’ was improved compared to 2018. However, based on the applied sampling strategy and the low number of evaluated colonies, it is not possible to draw conclusions on the consistency of observed repeatabilities over the years, as only 29 to 47 colonies were evaluated for each trait during these three years. In order to get more reliable values, repeatability should be calculated across multiple years based on data recorded at constant dates annually. This would facilitate the identification of suitable trait evaluation periods during which the measurements are more repeatable. Low repeatability was also reported for other resistance traits, e.g., hygienic behaviour towards dead brood [28,29]. Despite low repeatability, it has been demonstrated that this trait can be improved by selection [30,31,32]. As it has previously been suggested that measurements for several traits can be repeated to obtain more robust values [33,34], this could also be applied for ‘*Recapping*’ and ‘*Solidness*’.

Heritability of ‘*Recapping*’ was as low as the previous reported estimates for recapping measured by the cell-by-cell inspection protocol [35], whilst the heritability estimate of ‘*Solidness*’ was compatible with selection. However, for both traits, more data is needed to obtain more precise estimates.

The two new traits could not be linked to better colony resistance against *V. destructor*. An association between ‘*Recapping*’ and hygienic behaviour is suggested from our data; this is comparable with the published correlation between hygienic behavior and recapping data measured by the previously developed protocol [35]. This association could therefore be investigated in more detail by a direct comparison of both methods, should they provide similar rankings of evaluated colonies, to determine whether or not they indeed refer to the same trait. Past studies [27,35], described an association between recapping and *V. destructor* infestation levels, which we did not observe in our analysis. It can be hypothesised that both traits either have a distinct genetic background or that the best periods to measure them do not overlap. In this study, it was not possible to evaluate the mite infestation rate and the number of mite offspring in recapped cells. The commonly applied protocol to evaluate recapping is more appropriate for this, as it enables cell-by-cell content analysis. ‘*Solidness*’ is associated with colony size, as colonies rearing more compact brood have more brood and so more workers. However, even within large colonies, which can host more mites, direct associations between ‘*Solidness*’ and *V. destructor* infestation levels were not found. It therefore seems that ‘*Solidness*’ is not directly associated with *V. destructor* resistance but could be useful for selecting honey bees that will build up larger colonies.

Compared to previous results based on performance testing carried out by beekeepers [18], some traits show higher heritability in our experimental populations. For instance, *V. destructor* infestation levels showed moderate heritability estimates in our experimental population, whilst the estimates were very low or equalled zero using the observations from beekeepers. Infestation level in summer had moderate heritability values between 0.5 and 0.7, which seems promising for selection. However, the infestation growth rate correlates better with the infestation level in spring (−0.64) than with the infestation level in summer (0.38). Furthermore, these associations demonstrate that the assessment of *V. destructor* infestation in spring is not practical for later identifying colonies with lower mite development [27]. This may explain the low heritability for the infestation growth rate (0.13 to 0.15), which has also been identified by other studies [8]. Thus, the obtained heritability values for infestation levels in summer may result from yet-unidentified particularities of the dataset. Infestation level in summer was correlated with hygienic behaviour: colonies being slow at evacuating dead brood (‘*Hygfull*’) also had more mites at this time of the year (r = 0.32). As the association between hygienic behaviour and infestation level is still being discussed in the literature [9,10,11], more data is needed to better determine the efficacy of hygiene for reducing mite infestation levels.

Estimating genetic parameters in small populations can be challenging, as obtained estimates often have high standard errors [29,36]. However, small-scale trials are required to develop new traits [29] and to verify the relevance of a trait used by beekeepers under controlled conditions [28]. The size of experimental populations is either limited by the size of the population from which they derive, by the capacity of the research institute, or by the duration of funding. In the current literature, a high proportion of previously reported heritability estimates were computed based on less than 100 colonies [36,37,38,39,40,41,42,43]. Such results can be strongly biased and misleading. Therefore, this analysis presents preliminary results that can be decisive for selection but that will need to be confirmed after more years of data collection.

The validation of the heritabilities and assertions between traits found in our study require a larger testing capacity in the field, with an increased number of different environmental conditions and more precise trait assessment. This is necessary for beekeepers to evaluate the relevance of given traits under field conditions, as beekeepers’ management may differ from beekeeping performed by scientists. Swiss beekeepers currently involved in selection of *A. m. mellifera* do their best to assess different traits, but the selected population is itself of limited size, and the association faces difficulties in recruiting motivated members for colony evaluation. Therefore, an increased dataset will require the allocation of testing apiaries in different environments and a dedicated workforce able to test several hundred colonies each year based on scientific protocols, an undertaking that requires a financial investment far beyond either the framework of ordinary research projects or the capacity of one beekeeping association. Such problems may be encountered by other countries where honey bee populations are also of small size or where beekeepers are not yet coordinated around shared selection programmes.

## Figures and Tables

**Table 1:** Traits recorded in the framework of the colony performance testing protocol at the experimental apiaries.

Variable Name	Trait	Unit	N	Min Median Max	Evaluation Method	Frequency	Data Aggregation Method
*‘Recapping’*	Recapping of worker brood cells	% of recapped cells	115	0 0.8 28.1	Sample of cell caps cut from a brood area next to emerging bees (see Figure 1a)	Every three weeks from spring to summer	Mean of repetitions
*‘Solidness’*	Compactness of brood	Number of cells	121	50.5 54.8 107.5	Number of cells between first and last pinned cells counted when pin-test performed on 50 capped cells (see Figure 1b)		
*‘Honey’*	Honey production	Kg of extracted honey	97	0 17.9 60.1	Weighing of honey combs before extraction; deduction of the weight of the empty combs.	At each harvest	Sum of all harvests during evaluation period
*‘Gentleness’*	Gentleness	Score between 1 (not gentle) and 4 (very gentle)	111	1 2.7 3.7	According to Smartbees testing protocol [20]	Every 3 weeks between spring and summer	Mean of all notes
*‘Calmness’*	Calmness	Score between 1 (not calm) and 4 (very calm)	110	1 2.5 3.5			
*‘Swarming’*	Swarming	Score given by evaluator	106	0 12 38	Colony gets 1 if presence of queen cells with egg, 2 if presence of queen cells with larvae, 3 if presence of capped queen cells.	At each visit (up to 2 times/week during swarming season)	Sum of scores of all visits. Colonies which swarmed got a final score of (maximal score of the apiary) +1.
**Variable Name**	**Trait**	**Unit**	**N**	**Min** **Median** **Max**	**Evaluation** **Method**	**Frequency**	**Data Aggregation Method**
*‘Varroaspring’*	Varroa rate in spring	Naturally fallen mites per day in Spring	112	0 1 51	Naturally fallen mites counted on an oiled paper placed below the meshed floor of the hive; bi-weekly counts and paper replacement; total timeframe of 3 weeks	During the three first weeks of testing in Spring	Mean mite fall per day
*‘Varroasummer’*	Varroa rate in summer	Mites/100 adult worker bees	93	0 1.3 15.2	Sample of about 300 adult workers taken from brood frames, washed with soap water	Once, at the end of the evaluation season	-
*‘Varroacumul’*	Varroa cumulated mite fall	Naturally fallen mites	92	7 171 2559	Naturally fallen mites counted on an oiled paper placed below the colony; bi-weekly counts and paper replacement;	During the whole season	Sum of all counted mites
*‘Varroagrowth’*	Varroa growth rate between Spring and summer	-	91	0.52 1.13 2.06	-	-	Combination of mite fall in Spring and infestation rate on workers in summer according to [21]
**Variable Name**	**Trait**	**Unit**	**N**	**Min** **Median** **Max**	**Evaluation** **Method**	**Frequency**	**Data Aggregation Method**
*‘Hygfull’*	Number of non-opened cells at the end of the pin-test	% of non-opened cells	121	0 22.8 96.3	Pin-killed brood according to standard protocol [19] checked after having been exposed to workers during 12 h overnight	Every three weeks from spring to summer	Mean of repetitions
*‘Hygprogress’*	Number of cells containing pupae in progress of being removed at the end of the pin-test	% of cells with pupae in progress of being removed	121	2.7 34.4 62.0			
*‘Hygempty’*	Number of completely cleared cells at the end of the pin-test	% of completely cleared cells	121	1 36.4 96.0			
*‘Colbeesspring’*	Colony size (workers) in spring	Number of workers	116	3800 11,100 22,600	Estimation by Liebefeld method [22,23]	Once at first colony evaluation in spring	-
*‘Colbroodspring’*	Colony size (brood) in spring	Surface of brood in dm^2^	116	9.7 50.9 105.9			
*‘Colbeessummer’*	Colony size (workers) in summer	Number of workers	102	6100 13,900 20,600		Once at last colony evaluation in summer	-
*‘Colbroodsummer’*	Colony size (brood) in summer	Surface of brood in dm^2^	102	14.9 59.3 106.5			
*‘Colbeesgrowth’*	Colony size (workers) growth rate from spring to summer	-	97	0.50 1.31 3.12	Ratio of nb of workers in summer on nb of workers in spring	-	-
*‘Colbroodgrowth’*	Colony size (brood) growth rate from spring to summer	-	97	0.58 1.12 3.65	Ratio of brood surface in summer on brood surface in spring	-	-

**Table 2:** Repeatability values and associated standard errors (between brackets) for *‘Recapping’* and *‘Solidness’* for measurements (repetitions A to F) performed during years 2018, 2019, and 2020.

Recapping						
**2018**		**R2018-B**	**R2018-C**	**R2018-D**	**R2018-E**	**R2018-F**
	**R2018-A**	0.01 (0.22)	0.21(0.21)	−0.05 (0.22)	0.58 (0.15)	0.00 (0.22)
	**R2018-B**		0.34(0.20)	−0.03 (0.22)	0.41 (0.19)	−0.13 (0.22)
	**R2018-C**			0.07 (0.22)	0.59 (0.15)	0.19 (0.22)
	**R2018-D**				−0.07 (0.22)	−0.23 (0.21)
	**R2018-E**					0.46 (0.18)
**2019**		**R2019-B**	**R2019-C**			
	**R2019-A**	0.31 (0.17)	0.32 (0.17)			
	**R2019-B**		0.29 (0.17)			
**2020**		**R2020-B**	**R2020-C**	**R2020-D**	**R2020-E**	
	**R2020-A**	0.28 (0.16)	0.08 (0.17)	0.02 (0.17)	−0.04 (0.17)	
	**R2020-B**		0.15 (0.17)	0.15 (0.17)	0.07 (0.17)	
	**R2020-C**			0.44 (0.14)	0.22 (0.16)	
	**R2020-D**				0.05 (0.17)	
**Solidness**						
**2018**		**S2018-B**	**S2018-C**	**S2018-D**	**S2018-E**	**S2018-F**
	**S2018-A**	0.59 (0.15)	0.18 (0.22)	0.19 (0.22)	−0.19 (0.22)	0.57 (0.15)
	**S2018-B**		−0.07 (0.22)	−0.06 (0.22)	−0.29 (0.21)	0.13 (0.22)
	**S2018-C**			0.18 (0.22)	0.19 (0.22)	0.09 (0.22)
	**S2018-D**				0.06 (0.22)	−0.02 (0.22)
	**S2018-E**					0.09 (0.22)
**2019**		**S2019-B**	**S2019-C**	**S2019-D**		
	**S2019-A**	0.12 (0.19)	−0.01 (0.19)	0.11 (0.19)		
	**S2019-B**		0.49 (0.15)	0.23 (0.18)		
	**S2019-C**			0.35 (0.17)		
**2020**		**S2020-B**	**S2020-C**	**S2020-D**	**S2020-E**	
	**S2020-A**	0.21 (0.17)	0.01 (0.17)	0.13 (0.17)	0.46 (0.14)	
	**S2020-B**		0.42 (0.14)	0.80 (0.06)	0.53 (0.13)	
	**S2020-C**			0.46 (0.14)	0.37 (0.15)	
	**S2020-D**				0.73 (0.08)	

**Table 3:** Heritabilities (diagonal, grey) for traits and phenotypic correlations (off-diagonal) corrected for apiary effects and associated standard errors (between brackets). For explanation of traits see Table 1. Heritabilities were estimated by the model on worker effects (upper value) and the model of queen effects (lower value). Pearson’s correlation coefficients between phenotypes corrected for apiary effects were estimated by the model on worker effects. Bold correlation coefficients significantly (*p* < 0.05) differed from 0; green background corresponds to a positive correlation, an orange background to a negative correlation.

	*Recapping*	*Solidness*	*Honey*	*Calmness*	*Swarming*	*Varroaspring*	*Varroasummer*	*Varroacumul*	*Varroagrowth*	*Hygfull*	*Hygprogress*	*Hygempty*	*Colbeesspring*	*Colbroodspring*	*Colbeessummer*	*Colbroodsummer*	*Colbeesgrowth*
*Recapping*	*0.05 (0.24)* *0.04 (0.16)*																
*Solidness*	−0.14 (0.11)	*0.25 (0.29)* *0.24 (0.26)*															
*Honey*	−0.01 (0.11)	−0.01 (0.11)	*0.40 (0.28)* *0.35 (0.27)*														
*Calmness*	−0.08 (0.11)	0.11 (0.11)	0.12 (0.11)	*0.62 (0.28) * *0.54 (0.31)*													
*Swarming*	0.04 (0.11)	−0.06 (0.11)	−0.09 (0.11)	−0.10 (0.11)	*0.32 (0.34)* * 0.17 (0.25)*												
*Varroaspring*	−0.01 (0.11)	−0.06 (0.11)	0.12 (0.11)	0.05 (0.11)	0.17 (0.11)	*0.25 (0.20)* * 0.05 (0.18)*											
	*Recapping*	*Solidness*	*Honey*	*Calmness*	*Swarming*	*Varroaspring*	*Varroasummer*	*Varroacumul*	*Varroagrowth*	*Hygfull*	*Hygprogress*	*Hygempty*	*Colbeesspring*	*Colbroodspring*	*Colbeessummer*	*Colbroodsummer*	*Colbeesgrowth*
*Varroasummer*	−0.19 (0.11)	0.11 (0.11)	−0.08 (0.11)	0.07 (0.11)	0.09 (0.11)	**0.29** **(0.10)**	*0.49 (0.26) * *0.72 (0.30)*										
*Varroacumul*	−0.07 (0.11)	−0.09 (0.11)	0.02 (0.11)	0.13 (0.11)	0.03 (0.11)	**0.38** **(0.09)**	**0.47** **(0.09)**	*0.16 (0.23)* * 0.08 (0.18)*									
*Varroagrowth*	−0.11 (0.11)	0.13 (0.11)	−0.09 (0.11)	0.04 (0.11)	−0.15 (0.11)	**−0.64** **(0.06)**	**0.38** **(0.09)**	−0.04 (0.11)	*0.15 (0.30) * *0.13 (0.24)*								
*Hygfull*	**−0.23** **(0.10)**	0.15 (0.11)	−0.08 (0.11)	0.03 (0.11)	−0.02 (0.11)	0.04 (0.11)	**0.32** **(0.10)**	**0.25** **(0.10)**	0.10 (0.11)	*0.60 (0.18)* * 0.52 (0.20)*							
*Hygprogress*	0.11 (0.11)	0.00 (0.11)	−0.05 (0.11)	0.09 (0.11)	0.08 (0.11)	0.12 (0.11)	−0.14 (0.11)	0.04 (0.11)	−0.14 (0.11)	**−0.37** **(0.09)**	*0.20 (0.22) * *0.11 (0.15)*						
*Hygempty*	0.20 (0.11)	−0.16 (0.11)	0.12 (0.11)	−0.08 (0.11)	−0.04 (0.11)	−0.10 (0.11)	**−0.26** **(0.10)**	**−0.29** **(0.10)**	−0.03 (0.11)	**−0.85** **(0.03)**	−0.17 (0.11)	*0.47 (0.21)* * 0.38 (0.20)*					
	*Recapping*	*Solidness*	*Honey*	*Calmness*	*Swarming*	*Varroaspring*	*Varroasummer*	*Varroacumul*	*Varroagrowth*	*Hygfull*	*Hygprogress*	*Hygempty*	*Colbeesspring*	*Colbroodspring*	*Colbeessummer*	*Colbroodsummer*	*Colbeesgrowth*
*Colbeesspring*	0.12 (0.11)	**−0.24** **(0.10)**	**0.29** **(0.10)**	0.05 (0.11)	**0.41** **(0.09)**	**0.33** **(0.10)**	−0.05 (0.11)	0.14 (0.11)	**−0.36** **(0.10)**	0.01 (0.11)	0.09 (0.11)	−0.06 (0.11)	*0.11 (0.22)* * 0.06 (0.15)*				
*Colbroodspring*	0.06 (0.11)	−0.07 (0.11)	**0.34** **(0.10)**	**0.10** **(0.11)**	**0.26** **(0.10)**	**0.36** **(0.10)**	−0.01 (0.11)	0.10 (0.11)	**−0.36** **(0.10)**	−0.09 (0.11)	**0.24** **(0.10)**	−0.04 (0.11)	**0.65** **(0.06)**	*0.10 (0.21)* * 0.08 (0.16)*			
*Colbeessummer*	−0.03 (0.11)	**−0.28** **(0.10)**	**0.40** **(0.09)**	0.14 (0.11)	0.05 (0.11)	0.09 (0.11)	**0.28** **(0.10)**	**0.31** **(0.10)**	0.10 (0.11)	**0.36** **(0.10)**	−0.16 (0.11)	**−0.29** **(0.10)**	**0.23** **(0.10)**	**0.23** **(0.10)**	*0.71 (0.19)* * 0.65 (0.24)*		
*Colbroodsummer*	0.02 (0.11)	**−0.26** **(0.10)**	**0.23** **(0.10)**	0.00 (0.11)	0.10 (0.11)	0.14 (0.11)	−0.02 (0.11)	0.21 (0.10)	−0.14 (0.11)	**−0.22** **(0.10)**	0.17 (0.11)	0.14 (0.11)	**0.26** **(0.10)**	**0.38** **(0.09)**	**0.41** **(0.09)**	*0.01 (0.20) * *0.07 (0.18)*	
*Colbeesgrowth*	−0.12 (0.11)	−0.07 (0.11)	−0.14 (0.11)	−0.06 (0.11)	**−0.32** **(0.10)**	**−0.24** **(0.10)**	0.20 (0.11)	0.01 (0.11)	**0.38** **(0.09)**	0.18 (0.11)	−0.08 (0.11)	−0.15 (0.11)	**−0.69** **(0.06)**	**−0.51** **(0.08)**	**0.38** **(0.09)**	0.08 (0.11)	*0.19 (0.25)* * 0.12 (0.19)*

## Data Availability

The data that support the findings of this study are available from the corresponding author upon reasonable request.
